# LoRa Sensor Network Development for Air Quality Monitoring or Detecting Gas Leakage Events

**DOI:** 10.3390/s20216225

**Published:** 2020-10-31

**Authors:** Ernesto González, Juan Casanova-Chafer, Alfonso Romero, Xavier Vilanova, Jan Mitrovics, Eduard Llobet

**Affiliations:** 1 Electronic Engineering, Uiversitat Rovira i Virgili, MINOS, 43007 Tarragona, Spain; ernesto.gonzalez@urv.cat (E.G.); juan.casanova@urv.cat (J.C.-C.); alfonso.romero@urv.cat (A.R.); eduard.llobet@urv.cat (E.L.); 2 JLM Innovation GmbH, 72070 Tubingen, Germany; jan.mitrovics@jlm-innovation.de

**Keywords:** AQM, IoT, LoRa, WSN, graphene

## Abstract

During the few last years, indoor and outdoor Air Quality Monitoring (AQM) has gained a lot of interest among the scientific community due to its direct relation with human health. The Internet of Things (IoT) and, especially, Wireless Sensor Networks (WSN) have given rise to the development of wireless AQM portable systems. This paper presents the development of a LoRa (short for long-range) based sensor network for AQM and gas leakage events detection. The combination of both a commercial gas sensor and a resistance measurement channel for graphene chemoresistive sensors allows both the calculation of an Air Quality Index based on the concentration of reducing species such as volatile organic compounds (VOCs) and CO, and it also makes possible the detection of NO_2_, which is an important air pollutant. The graphene sensor tested with the LoRa nodes developed allows the detection of NO_2_ pollution in just 5 min as well as enables monitoring sudden changes in the background level of this pollutant in the atmosphere. The capability of the system of detecting both reducing and oxidizing pollutant agents, alongside its low-cost, low-power, and real-time monitoring features, makes this a solution suitable to be used in wireless AQM and early warning systems.

## 1. Introduction

According to the World Health Organisation, in 2016, household and ambient air pollution were responsible for seven million deaths [1]. During the last decade, many researchers have investigated both indoor and outdoor air quality monitoring systems because of air quality being intrinsically linked to human health [2,3,4,5,6] and the occurrence of premature deaths [7,8,9,10]. Therefore, having widespread, unattended portable and connected devices and networks for air quality monitoring and pollutant detection would be a decisive step forward for decreasing the prevalence of lethal diseases such as ischemic heart disease, stroke, chronic obstructive pulmonary disease, or even lung cancer [11,12,13]. According to the United States Environmental Protection Agency, people spend 90% or even more of their time in indoor environments, on average. Indoors, the concentration of some air pollutants can often be two to five times higher than the concentrations found outdoors [14]. The combustion of fossil fuels, gas stoves, tobacco smoke, and water heating systems that burn natural gas, cleaning supplies, paints, and insecticides, among others, are the principal sources of pollutant gases present indoors such as NO_X_, CO, and volatile organic compounds (VOCs) [14,15,16,17]. Outdoor air pollutants entering the buildings and those generated indoors are directly affecting human health since these can cause headache, hypoxia, or problems in vital systems such as the respiratory, cardiovascular, or central nervous [16,17,18]. Thus, indoor air quality monitoring is an important factor in enhancing the quality of health and comfort.

The conventional and most common techniques for air quality monitoring consist of stationary stations equipped with different instrumental techniques or manual sampling at different locations followed by analysis in specialised laboratories. These techniques can be very accurate but require the use of bulky, power-hungry, and expensive instruments [19,20]. Thus, these approaches do not provide a scalable solution for monitoring with enough spatial resolution air pollution events related to automotive combustion or burning fuels for cooking in urban scenarios [20,21,22,23]. Furthermore, these technologies demand trained personnel to operate with complex equipment and, obviously, they cannot be implemented in portable, low-cost, and low-power devices. Different sensors employing diverse operating principles, such as electrochemical [4,24], non-dispersive infrared [25,26], and chemoresistive have been considered for devising air quality monitoring systems at lower costs [27,28]. These can be employed to develop portable analysers, which can be endowed with low power (at least in comparison to bulky instrumental techniques), communication ability with local or cloud servers, and the capability to detect alarm situations locally. Among inexpensive sensors, metal oxide (MOX) chemoresistors have the advantage of being easily miniaturised and exhibiting high sensitivity in the detection of air pollutants such as VOCs, NOx, or CO. However, MOXs show significant cross-sensitivity issues, and they generally require being operated at high temperatures (up to 500 °C) which compromise their usefulness in portable applications, where low-power consumption features are needed [19,29,30]. Conversely, chemoresistors employing carbon nanomaterials (graphene and carbon nanotubes) have been suggested as an alternative to detect low concentrations of some air pollutants at ppb to ppm levels. These nanomaterials present favourable electronic properties and can be easily modified or functionalised to reach remarkable sensitivity and acceptable selectivity towards air pollutants [31,32,33]. In addition, carbon nanomaterial chemoresistors can be operated slightly above or even at room temperature, which allows reducing power consumption in portable solutions, leading to low-power devices [32,34].

In the last few years, several authors have reported the use of Wireless Sensor Networks (WSNs) for air quality monitoring systems [20,22]. In that sense, some decision-making systems have been designed using both Local Area Networks (LANs) for the monitoring of local air quality or generating an alarm while under a gas pollution event [35,36] and Wide Area Networks (WANs) for remote control and monitoring [37,38,39]. Many WSNs have been deployed to cover a broad range of applications such as precision agriculture [40,41,42], healthcare [43,44,45], military industry [46,47,48], and air quality monitoring [22,24,25]. The application fields for WSNs are becoming wider every day. The development of the Internet of Things (IoT) paradigm has fuelled the implementation of WSNs connected to cloud server services with monitoring, data analysis, or data processing capabilities [39,49,50,51]. The IoT encompasses smart devices and sensors that are able to communicate with one each other, being accessible at any moment, everywhere. The connectivity is the main requirement for IoT, and its applications support a varied set of devices and communication protocols, from sensors to powerful back-end servers with data processing capabilities [52,53]. Many IoT systems and WSNs deployed during the last few years have employed some well-known devices and communication protocols such as radiofrequency [49,54], Global System for Mobile Communications (GSM) [20,55], Wi-Fi [39,56], or ZigBee [57]. However, most of these technologies present some disadvantages under certain conditions, including large power usage, limitations in transmission distance, or security issues. To overcome the limitations presented by the above-mentioned wireless technologies, low-power wide area networks (LPWANs) have emerged allowing long-range wireless communications at a low bit rate using low-power consumption [39,50,58,59]. Figure S1 in the supporting information shows the growth of the research areas for which this paper is relevant, which is reflected as the number of publications appearing in Scopus between years 2010 and 2020 related to IoT, LoRa, and air quality monitoring.

Among the promising technologies used in the LPWAN space are Narrowband Internet of Things (NB-IoT), Sigfox from Sigfox, Labège, France, and LoRa. While NB-IoT presents the largest data rate and payload size of these three technologies, it operates in licensed Long-Term Evolution (LTE) frequency bands. Thus, the use of NB-IoT was not considered to keep costs low. The Sigfox presents a higher transmission range than LoRa (3–10 km for Sigfox and 2–5 km for LoRa, in urban areas), but Sigfox is deployed by network operators, so users need to pay subscription charges for every node installed. Moreover, Sigfox transmits messages multiple times to improve reliability, resulting in high energy consumption. Hence, LoRa and LoRaWAN protocol were selected to develop the network [39,50,58,59].

LoRa is a spread spectrum modulation technique derived from chirp spread spectrum (CSS) technology. This physical layer technology works in the unlicensed sub-GHz industrial, scientific, and medical (ISM) band. Depending on the spreading factor (SF) and the channel bandwidth (BW) settings, the data rate supported by LoRa varies from 300 bps to 50 kbps. The transmission power (TP), SF, coding rate (CR), and preamble length define the signal airtime and power consumption [59].

LoRaWAN is a media access control (MAC) protocol for wide area networks designed to allow low-powered devices to communicate with Internet-connected applications over long-range wireless connections. Innovative LoRaWAN features include support for redundant operation, geolocation, low-cost, and low-power applications. Devices can even run on energy harvesting technologies (i.e., means of collecting energy from the environment used to power devices or to extend the system battery lifetime) enabling the mobility and ease use of IoT. LoRaWAN protocol provides origin authentication, integrity protection, replay protection, and full end-to-end encryption [59,60]. The use of LoRa for the design of the nodes and the network communication allows developing low-power nodes with long data transmission distance. The LoRaWAN protocol ensures the security of the data by the authentication process between nodes and the network server and the end-to-end messaging encryption.

The aim of the present paper is to discuss the development of an IoT implementation for inexpensive indoor air quality monitoring (e.g., at train, bus stations, shopping centres, or theatres) and real-time pollutant detection. This consists of a wireless sensing network in which its sensing nodes include both commercial and lab-made (presented in Section 3) chemoresistive sensors. The network was deployed at the University of Tarragona science campus for testing and validation purposes. Within a sensing node, the commercially available sensor allows estimating an air quality index, which relates to the concentration of total VOCs present in the node environment. The lab-synthesised sensor enables the detection of a pollutant, nitrogen dioxide (NO_2_), released to the atmosphere mainly from automotive exhausts [61] and uses graphene nanoplatelets as gas-sensitive material. Previous works have already demonstrated the usefulness of graphene for detecting NO_2_ at trace levels [62,63]. Furthermore, their capability for being operated at room temperature makes graphene sensors very attractive for developing low-power applications. This sensor network is meant to be used indoors to monitor air quality and identify sudden changes in the background concentration of some target pollutant species, raising an alarm. While the case of nitrogen dioxide is discussed here, the system is flexible enough to use sensors employing different gas sensitive materials, and thus, it can be adapted easily to the monitoring of different targets.

## 2. System Design and Implementation

### 2.1. Network Deployment

**Figure 1:**
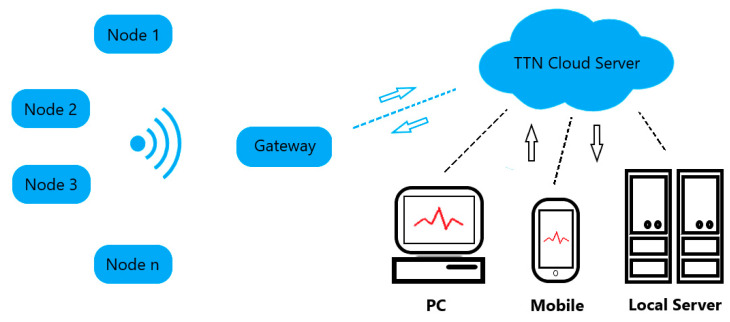
LoRa (short for long-range) sensor network operation scheme.

A LoRa-based, robust, low-power, and scalable wireless sensor network for air quality monitoring and pollutant detection is presented. This sensor network is physically composed by two main elements: the gateway and the end nodes, which have the sensors on them. Moreover, the network uses a cloud server for storing the data from the sensors, and the processing or visualisation of the data is made through a local server, a PC, or a mobile phone accessing a front-end application. The network deployment was carried out as follows: The indoor LoRa gateway was placed in a location where it was able to access the Internet through a Wi-Fi connection. Once connected to The Things Network (TTN) cloud server, the gateway was ready to forward the uplink and downlink messages sent from the nodes to the server and vice versa. Nodes perform measurements, codify the information, and send the data at every sampling period and then go to deep sleep mode. The data received in the cloud server is processed and stored for later visualisation. Front-end web and desktop applications were developed to access and visualise the data stored in the cloud server. Figure 1 depicts a scheme of the sensor network developed.

### 2.2. Gateway

The sensor network is devised for monitoring indoor air quality (nodes were distributed in different locations at the University campus), the gateway selected was The Things Indoor Gateway (TTIG) from The Things Industries. TTIG is a low-cost, fully compliant LoRaWAN gateway, which uses Wi-Fi as backhaul. It has a Semtech SX1308 LoRa chipset from Semtech Corporation, Irvine CA, USA, which presents a receiver sensitivity of −140 dBm and uses the 868 MHz frequency band (in the European Union). This indoor gateway has an integrated antenna and presents the Federal Communication Commission (FCC) and European Economic Area (CE) certifications. The software running on the gateway makes it act as a bridge between the LoRa nodes and TTN cloud server.

### 2.3. End Nodes Design and Programming

#### 2.3.1. Hardware

**Figure 2:**
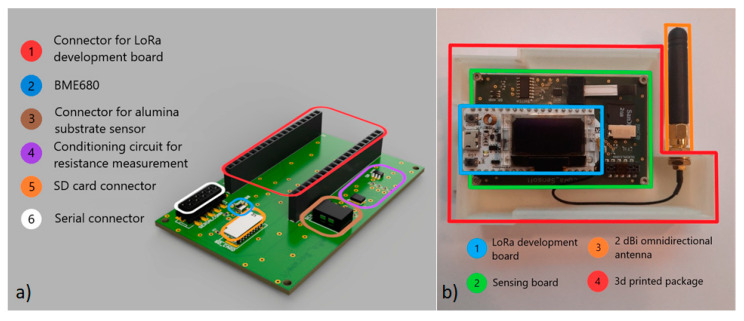
(**a**) Sensing board built for LoRa (short for long-range) nodes development and (**b**) end node without the front cover.

LoRa nodes (Figure 2b) developed in this project were conceived in two different but similar versions. Although both have sensing capabilities and are connected to the cloud server through the TTIG, one of those give the capacity of local visualisation of an air quality index through a display. This last was used for debugging and test purposes to compare the local data with the cloud data. End nodes built are formed by the following: (i) a LoRa development board, which includes the microcontroller unit and the LoRa module, (ii) the sensing board, (iii) a 2 dBi omnidirectional antenna, and (iv) a 3D designed and 3D-printed package.

**Figure 3:**
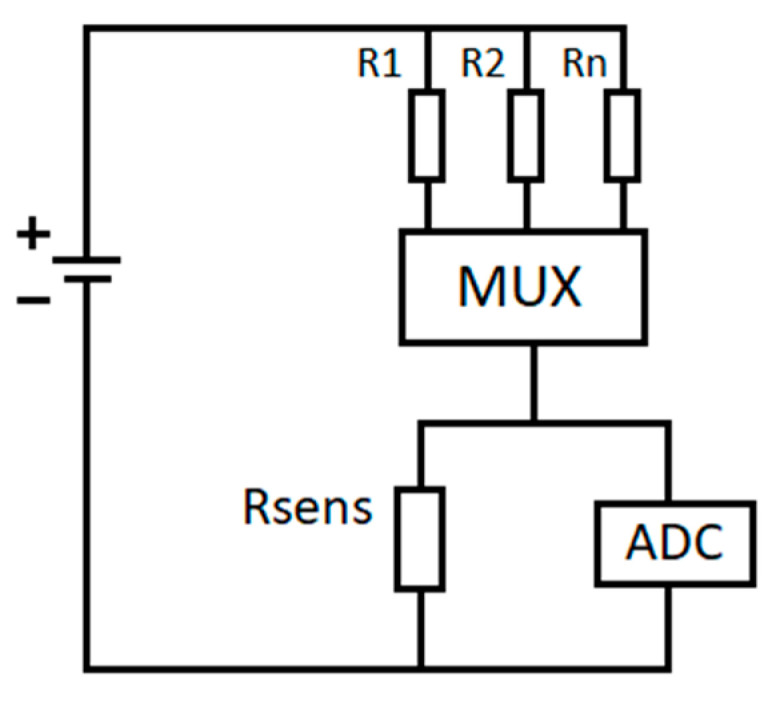
Voltage divider configuration used to measure the lab-made sensor resistance. Rsens represents the sensor resistance, which varies with the concentration of the target gas. R1, R2, and Rn are resistances with different and known values and are selected through the multiplexer (MUX) to keep the voltage measured by the analog-to-digital converter (ADC) in the maximum accuracy range.

The sensing board (Figure 2a) developed includes the following: (i) a connector for the LoRa development board, (ii) a 4 in 1 sensor BME680 from Bosch GmbH, Gerlingen, Germany, (iii) a connector for the lab-developed graphene sensor, (iv) a conditioning circuit for the readout of the graphene sensor, which consists of the voltage divider configuration shown in Figure 3, (v) a micro-SD card socket (for storing BME680 sensor state, LoRa activation keys and sensor measurements locally), and (vi) a general purpose serial connector (not used in this application).

The BME680 is a 4-in-1 digital low-power gas, pressure, temperature, and humidity sensor, which with the proper configuration and software libraries, presents a direct index for air quality (IAQ) output. The sensor has a V_DD_ main supply voltage that ranges from 1.2 to 3.6 V, which matches with the supply voltage of the microcontroller used. The IAQ value calculated using this sensor and the Bosch Sensortec Environmental Cluster (BSEC) library gives an indication of the total amount of VOCs and other reducing gases such as CO present in the studied atmosphere. The output is based on an intelligent algorithm that includes not only the resistance value of the metal oxide sensor the BME680 includes, but also humidity, temperature, and the measurement history values stored in the sensor state. The sensor state is stored after each measurement to be used in the sensor calibration for later IAQ value calculations. The sensor calibration typically encompasses up to 4 days of measurements. This index ranges between 0 and 500 (0 corresponds to the highest air quality and 500 corresponds to the worst air quality index) [64]. Some proceedings and conference papers have described the use of the BME680 for the development of indoor air quality monitoring portable and low-cost devices [65,66,67,68]. The measurement channel for the lab-made sensors is able to measure resistance values from few Ohms to approximately 3 MOhm, although this range could be easily adapted by changing the resistance values of the voltage divider configuration in the conditioning circuit. Although the performance of the resistance measurement is tested with the graphene sensor presented in Section 3, by selecting the sensors to be included in the node, the system can be used to cover different applications where the detection of a gas leakage is crucial. To help meet the low-power consumption features of the network, lab-made sensors are operated at room temperature. The nodes and gateway cost are shown in the supporting information.

#### 2.3.2. Software

**Figure 4:**
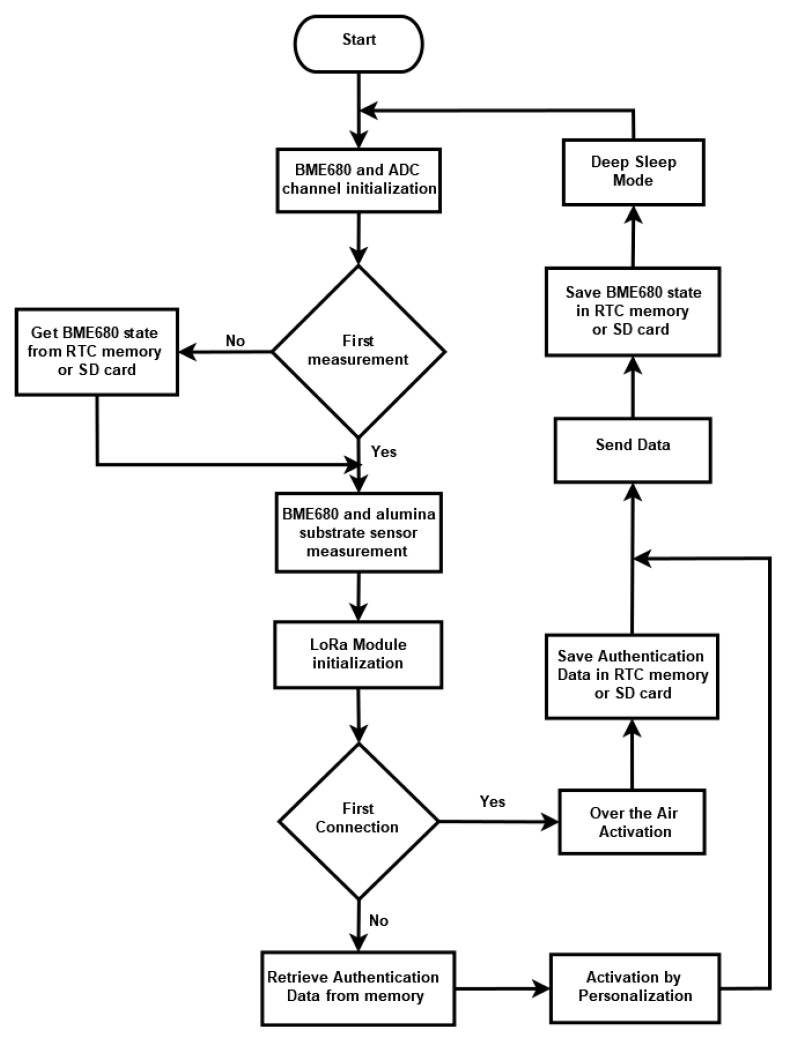
Flow diagram from the program running in the nodes.

The program running on the LoRa nodes was developed using C language in Arduino IDE from Arduino, Somerville, MA, USA. Once the nodes are powered up, reset, or woken up from deep sleep mode, the first step is to initialise the BME680 sensor and the ADC channel for the resistance measurements of the lab-made sensor. If the microcontroller unit (MCU) is reset or just powered up, the second step is to perform a measurement with the sensors. On the other hand, if the MCU wakes up from deep sleep mode, the previous state (see Figure 4 and hardware description section) of the BME680 sensor should be obtained from the real-time clock (RTC) memory or the SD card, because it will be used for the proper calculation of the IAQ value. When the initialisation of the BME680 sensor is finished, the measurements are performed. Once a measurement is completed, the third step is to initialise the LoRa module with the proper spreading factor and the transmission power to enable the network communication. The fourth step is to establish the connection with the server to send the data. If the node is connected for the first time to the server (after being powered up or reset), it performs a join procedure with the network cloud server through an Over the Air Activation, where a device address is assigned dynamically and security keys (Network Session Key and Application Session Key) are negotiated. After establishing the connection with the server, the device address and the security keys are stored for using them in further connections. Differently, if the MCU awakens from a deep sleep mode, an Activation by Personalisation is performed, using the authentication data stored in memory. In the fifth step, after establishing the connection, sensor data are codified in order to minimise the number of bytes uploaded to the server and then sent to the cloud server. In the sixth step, the BME680 sensor sate is saved in memory. Finally, in the seventh step, the node goes to deep sleep mode until the next sensor measurements. Figure 4 shows the flow diagram of the software running on the nodes.

### 2.4. TTN Application Server

Every uplink message that arrives to the TTN application server passes through a payload function in order to decode the data coming from the nodes. After the decoding, the sensor data are set in JSON format to match the requirements of the front-end application. The sensor data are stored through the Data Storage integration where it is kept available for 7 days to be downloaded through a Hypertext Transfer Protocol (HTTP) request.

### 2.5. Front-End Application

**Figure 5:**
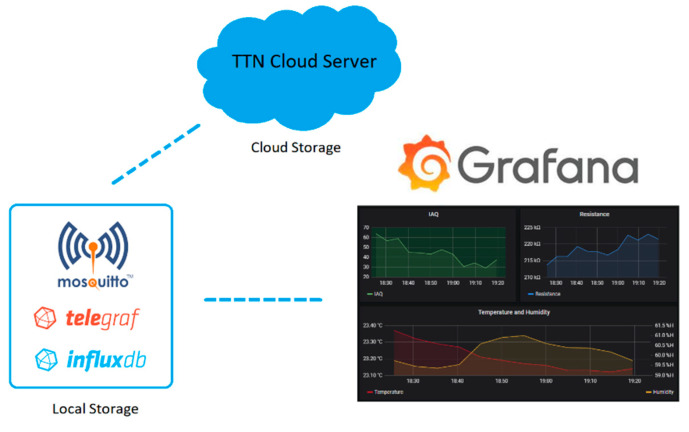
Sensor data path from The Things Network (TTN) cloud server to the front-end application.

For developing the front-end application, a set of Open-Source Software (OSS) was used, thus, saving money from the overall cost of the solution by avoiding licensing and software maintenance fees. The visualisation data solution presented in this section consists of a local server that stores the sensor data locally and makes it available to be visualised in a web service accessible from any device having internet access. The server could be deployed in any system running a Linux distribution (Ubuntu, Debian, or even Raspbian when running on a Raspberry Pi). Telegraf (from InfluxData Inc., San Francisco, CA, USA) is a server agent used to collect sensor data arriving to the TTN Message Queue Telemetry Transport (MQTT) broker through an MQTT consumer. With this purpose, a Mosquitto (from Eclipse Fundation, Ottawa, ON, Canada) client is set to connect with the TTN broker. The data collected by the Telegraf instance is stored in InfluxDB (from InfluxData), which is a time series database. For the visualisation of the data, Grafana was selected, which is an analytics and monitoring solution from Grafana Labs, New York, NY, USA. Figure 5 depicts the sensor data path from the TTN Cloud Server to the front-end application.

## 3. Graphene Sensor

### 3.1. Sensor Fabrication

A graphene-based gas sensor was fabricated by preparing a solution with 1 mg of graphene nanoplatelets from Strem Chemicals, Inc., Newburyport, MA, USA, dispersed in 10 mL of toluene. Afterwards, graphene nanoplatelets were exfoliated during one hour at high frequency (35 kHz) by using an ultrasonic bath (from Bandelin electronic GmbH, Berlin, Germany). Then, the solution was deposited onto the screen-printed platinum electrode area of an alumina substrate by the spray-coating technique. During this deposition, the substrate was placed in a hotplate at 125 °C in order to obtain a more homogeneous sensitive layer. Finally, the sensor was dried in an oven at 130 °C to ensure the complete removal of the solvent. A picture of the coated alumina substrate is shown in the supporting information (Figure S2).

### 3.2. Characterisation Techniques

The graphene used was characterised by several techniques, such as Raman spectroscopy, X-ray photoelectron spectroscopy (XPS), field-effect scanning electron microscope (FESEM), and high-resolution transmission electron microscopy (HR-TEM).

The graphene crystallinity was evaluated using a Raman spectrometer from Renishaw, plc. (Wotton-under-Edge, UK) coupled to a confocal Leica DM2500 microscope from Leica Microsystems GmbH, Wetzlar, Germany. The laser employed had a wavelength of 514 nm. The chemical composition of graphene was obtained by using an XPS spectrometer from SPECS GmbH, Berlin, Germany equipped with a non-monochromatic X-ray source (Al). Graphene morphology was studied using a FESEM and HR-TEM. In particular, graphene porosity and layer homogeneity were studied using a FESEM from Carl Zeiss AG, Oberkochen, Germany; meanwhile, graphene sheet sizes were observed via HR-TEM from Jeol Ltd., Tokyo, Japan.

### 3.3. Graphene Characterisation

According to the graphene manufacturer, this nanomaterial has a surface area of 750 m^2^/g and electrical conductivity of 10^7^ and 10^2^ S/m (parallel and perpendicular to its surface, respectively). Regarding the lateral size, different graphene nanoplatelets are stacked with an interplanar distance of 3.35 Å, while the overall graphene nanoplatelet aggregates have a thickness of a few nanometers.

**Figure 6:**
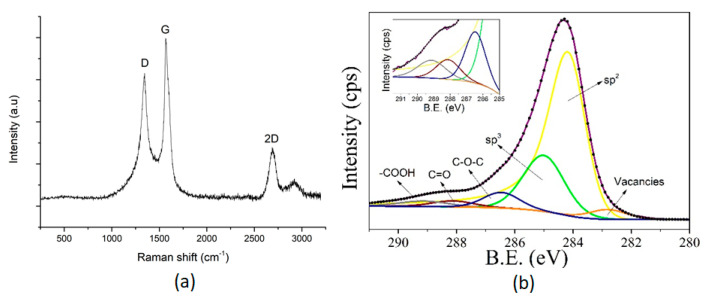
Raman spectra for the bare graphene (**a**). Deconvolution of the C1s core level peak for graphene (**b**). The inset shows the peaks related to oxygen functional groups.

A Raman analysis was conducted in order to study the graphene crystallinity. Figure 6a shows a significant D band at 1342 cm^−1^, which indicates the presence of defects (e.g., broken sp^2^ bonds, carbonaceous impurities, or amorphous carbon) in the crystalline structure. Conversely, the G band located at 1572 cm^−1^ reveals the stretching of C–C bonds, which are related to in-plane oscillations of sp^2^ configuration [69]. In consequence, the intensity ratio I_D_/I_G_ is commonly used as an indicator of the crystallinity in carbon nanomaterials [70]. In that sense, a value close to 0 reveals an insignificant number of defects (absence of I_D_ peak), being translated in highly crystalline graphene [71]. However, experimental data obtained from Figure 6a reveal an I_D_/I_G_ ratio of 0.79, indicating moderate graphene crystallinity. However, this fact is interesting from the gas sensing point of view, because these defects usually act as active sites for gas interaction and are also useful for further graphene functionalisation.

Additionally, an XPS was performed to obtain the elemental quantification. Regarding this, the graphene employed presents 89.99, 9.46 and 0.55% of carbon, oxygen and nitrogen, respectively. The fitting analysis of the C1s peak, centred at 284.4 eV, is represented in Figure 6b. This deconvolution shows a predominant peak at 284.2 eV, corresponding to the characteristic photoelectrons emitted from the graphitic-like carbon atoms. In other words, this peak reveals the predominance of sp^2^ carbon configuration. Nevertheless, according to the low crystallinity registered in the Raman analysis, a significant peak related to amorphous carbon or sp^3^ configuration can be found at 285.1 eV [72]. Furthermore, the inset shows three components associated with the presence of oxygen functional groups. Thus, the carbon–oxygen bonds observed at 286.1, 288.1, and 289.2 eV correspond to the C-O, C=O, and –COOH species, respectively [73]; meanwhile, the peak at 282.8 reveal structural graphene defects related with carbon vacancies [74]. Finally, the residual amount of nitrogen detected during the element quantification can be attributed to the presence of imine (NH) and amine (NH_2_) functional groups grafted at the graphene surface.

**Figure 7:**
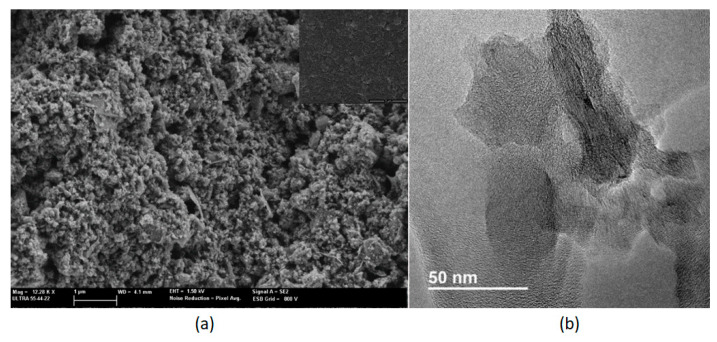
FESEM image showing the graphene sensor surface (**a**). HR-TEM image showing an example of the graphene layers size (**b**).

Images obtained by field-effect scanning electron microscope (FESEM) show a high porosity at the graphene surface (Figure 7a), which is helpful for gas sensing. In addition, the inset image (obtained at lower magnification) confirms that homogeneous layers can be obtained by using the spray coating technique. A high-resolution transmission electron microscopy (HR-TEM) analysis was also conducted (Figure 7b), showing graphene layers with diameters up to a hundred of nanometers.

### 3.4. Gas Sensing System

The characterisation of the gas sensing properties for the graphene sensor was performed in two different tests. First, laboratory conditions were set to test the sensor performance under fully controlled conditions, and next, real conditions were simulated to check its reliability for real-life applications. Results obtained from sensor characterisation tests run under laboratory conditions can be found in the supporting information.

The tests performed under close to real conditions were implemented using the graphene sensor connected to the LoRa node resistance measurement channel, which was placed in an acrylic chamber with an inner volume of about 4900 cm^3^. NO_2_ and CO calibrated cylinders of 1 ppm and 100 ppm, respectively (balanced in synthetic air), mixed with ambient air were used to set the desired gas concentrations. Gas flows were set by using a mass-flow controller system (EL-FLOW^®^) from Bronkhorst High-Tech, Ruurlo, Netherlands, and Flow View and Flow Plot software from the same company were employed, while a rotameter was used to control the ambient air flow. A total flow of 600 mL/min was kept across the chamber. The flow established was selected to simulate a gas diffusion process through the chamber avoiding the direct flow incidence on the sensors, which can influence the measurements. According to the inner volume of the acrylic chamber and the total flow through it, the time needed to renovate completely the atmosphere inside the chamber was about 8 min. Hence, the gas pulses implemented were programmed with a separation significantly higher than 8 min. Results from this test are shown and discussed in Section 4.2.

### 3.5. Gas Sensing Mechanisms

Pristine graphene, with its characteristic sp^2^ carbon configuration, is usually reported as a material with very poor sensitivity [75]. However, this limited responsiveness can be enhanced with further graphene functionalisation. In our case, Raman and X-Ray spectroscopy studies reveal a significant content of sp^3^ carbon configuration and oxygen functional groups. Thus, graphene nanoplatelets show significant sensitivity to nitrogen dioxide (NO_2_) [76]. This gas mainly interacts with the carbon lattice defects and oxygenated functional groups such as carbonyl or hydroxyl that act as reactive sites for adsorption of gases [77]. Therefore, gas molecules induce changes in the local carrier concentration when they are adsorbed on the graphene surface. In this regard, when a mild p-type semiconductor such as a graphene nanoflakes film is exposed to NO_2_ (an electron-withdrawing species), a decrease in the film resistance is recorded due to the increased concentration of positive charge carriers.

NO_2_ (gas) + e^−^ (surface) → NO_2_^−^ (ads) (1)

Considering the room temperature working conditions, the sensing mechanisms are probably ruled by the physisorption of gas molecules involving small charge transfers between nitrogen dioxide and the sensor surface. However, the slow baseline recovery indicates that the chemisorption of NO_2_ cannot be completely ruled out, which involves the exchange of higher amounts of charge between adsorbed gas species and the sensor surface (see Supporting Information).

In this regard, the physisorption of molecular oxygen (O_2_) due to the exposure of the sensitive film to air should be considered. Adsorbed atmospheric oxygen could eventually withdraw free electrons from the conduction band of graphene, resulting in an electron-depleted surface layer and the chemisorption of oxygen species [78]. Even so, isolated hydroxyl ions can be found at the sensor surface [76]. Thus, chemisorbed oxygen species can act as intermediate agents, catalysing the charge transfer processes between the sensor surface and gas molecules. Adsorbed oxygen species or isolated hydroxyl ions can be reactive with incoming oxidising agents such as NO_2_ [63,79]:

NO_2_ (gas) + O_2_^−^ (ads) + 2e^−^ (surface) → NO_2_^−^ (ads) + 2O^−^ (ads). (2)

Conversely, CO measurements reveal a lack of sensitivity for the bare graphene sensor. This agrees with theoretical calculations that report higher charge transfer (Q) upon adsorption for NO_2_ (0.19|e|) than for CO (0.01|e|) [80]. Nitrogen dioxide also shows higher adsorption energy (0.48 eV) in comparison to CO (0.12 eV) [81]. However, it is worth noting another additional effect, which is the essential role of the ambient moisture in the gas sensing performance. When both molecules, NO_2_ and H_2_O are simultaneously present, an additive effect is shown due to their electron-withdrawing behaviour over a mild p-type semiconductor such as graphene [82]. In other words, the NO_2_ response in humid atmosphere induces a larger increase in the conductivity. This fact is derived first from the charge transfer from graphene to water molecules when they are adsorbed in the sensor surface. Then, the subsequent water-mediated adsorption of NO_2_ can occur, further increasing the conductance change. This mechanism is especially worthy in measurements at room temperature. Indeed, Hall measurements have revealed that water molecules act as electron-withdrawing agents at room temperature [83], while CO behaves as an electron-donor gas. Hence, the opposite effects caused by H_2_O and CO molecules could significantly reduce the conductance changes recorded when these two analytes are present simultaneously. Nevertheless, the integration and combination of graphene sensors with other chemoresistive sensors such as metal oxides offer a great opportunity for better discriminating between electron donor and withdrawing gases.

## 4. Discussion

### 4.1. Network Performance

The LoRa sensor network was deployed in different locations at the University Campus with the purpose of testing the communication performance between different nodes and gateway configurations. Thus, the indoor gateway position was set in one location, while nodes were placed at different distances from the gateway, using different transmission powers. All the combinations used the rest of the network configuration parameters, namely carrier frequency (CF), BW, SF, CR and preamble length were set fixed. The CF used was 868 MHz, which is one of the two license-free sub-gigahertz radio frequency bands for Europe. The bandwidth was established in 125 kHz, thus ensuring enough bitrate and sensitivity for the detection of the signal. The spreading factor value of 7, which is the lowest possible, linked to a coding of 4/5 to allow the minimum time on air of the signal, and consequently a lower power consumption. The preamble length was also selected as the lowest possible value to guarantee the lowest power consumption. Table 1 depicts the Received Signal Strength Indicator (RSSI) and package loss rate. This last parameter was calculated as a rate between the number of received packages in the TTN cloud storage service and the theoretical amount of data sent by the nodes during 24 h.

During the test period (around 2 weeks for each pair TP and distance), the weather conditions were changing, and this is reflected in the package loss percentage shown in Table 1. Data from days with worst atmospheric conditions were not included in the package loss percentage calculation, as these values were far from the mean value. In cloudy, rainy, or stormy weather, the package lost rate reached between 14% and 25%.

**Figure 8:**
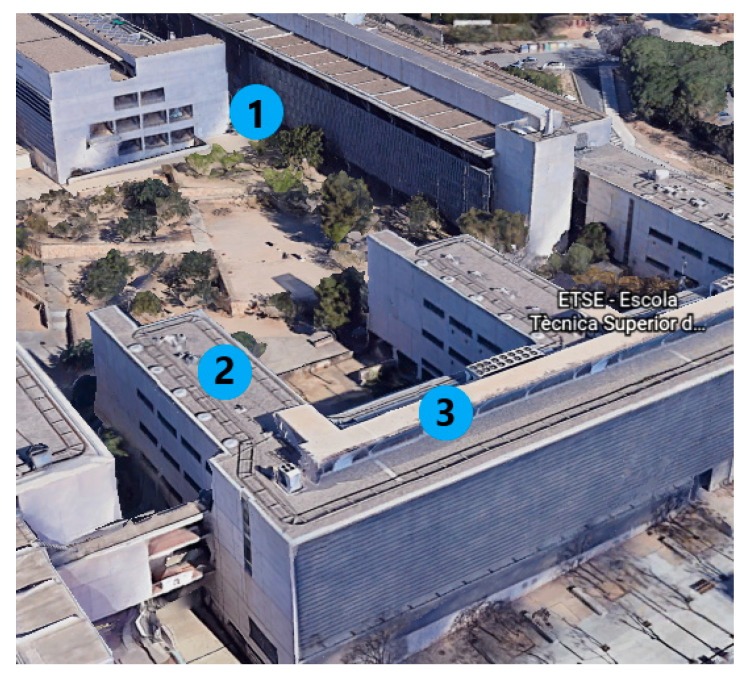
Location of the (1) gateway and (2–3) nodes in the second configuration. Gateway was placed at an office behind the metallic wall appearing in point 1 at ground level, while nodes were located at offices at points 2 (115 m from the gateway) and 3 (130 m from the gateway) in the third floor. Image taken from Google Earth.

Locations were selected according to two different scenarios. In the first one, the nodes were placed near the gateway, which were separated one from each other by several offices inside the same building. This configuration allows avoiding interferences provoked by unfavourable weather conditions, while the transmission power used can remain low, thus saving power without affecting the network performance. In the second location, the nodes were placed at 115–130 m away from the gateway (in different buildings), with non-straight sight and structural obstacles between them. This ensures the analysis of the network performance when conditions are not the most favourable for the communication. The transmission power was set at 12 dB, 14 dB, and 20 dB to compare the RSSI and package loss values obtained when the TP increases. Figure 8 depicts the location of gateway and nodes at the University Campus for the second configuration.

All RSSI values obtained for the pairs distance–transmission power were higher than the sensitivity of the gateway. For nodes placed in the same building, the package loss rate was less than 1% using a TP of 12 dB. This means just 1 or 2 package losses during 24 h of measurements in the worst case. For longer distances, the TP was increased from 12 to 20 dB (which is the maximum possible) to compare the performance of the network. Referring to Figure 8, the node located in point 2 was set with TP values of 12 and 14 dB. The RSSI and package loss values slightly improved when the transmission power increased. At position 3, nodes were deployed with a TP value of 20 dB to test the performance, when this is the maximum power value for the LoRa transceiver, and the location is the less favourable for the communication. In this case, the RSSI value slightly worsened, while the package loss percentage improved a bit.

Although nodes have been powered up through a standard wall socket, all the design and configuration characteristics were considered to use the nodes in a low-power mode. This enables powering up the nodes employing batteries. Using a TP of 12 dB and the rest of transmission parameters as mentioned above, the average power consumption for 1 h is about 1 mA. Thus, considering the use of a 2200 mAh battery, and assuming a total discharge of 85%, the estimated battery lifetime would be about 75 days.

The network is fully scalable (i.e., new nodes can be added) without the need of modifying any parameter on the gateway side. Thus, after the first deployment, the gateway can run continuously, and just each new node should be registered on the cloud server in order to assign the unique authentication parameters it needs to become part of the network. This allows enlarging the number of nodes at any time without affecting the global network performance.

### 4.2. Gas Sensing Performance

Both commercial (BME680) and lab-made (graphene) sensors where placed in the LoRa node and exposed to gas pulses of CO or NO_2_ at concentrations near the threshold limit value (TLV). With that, it was possible to simulate events of gas leakage or sudden increases in concentration when the concentration changes from the background level of the target species in the atmosphere to values above the TLV established by the European US agencies [84,85]. For this purpose, during the CO pulses, the concentrations were set at 35 and 50 ppm, while the NO_2_ concentration was set at 200 ppb. Pulse duration randomly varied between 5 and 15 min. As measurements were performed under real conditions, the relative humidity (RH) and temperature were variable during the test period, with average values about 50% and 30 °C, respectively.

**Figure 9:**
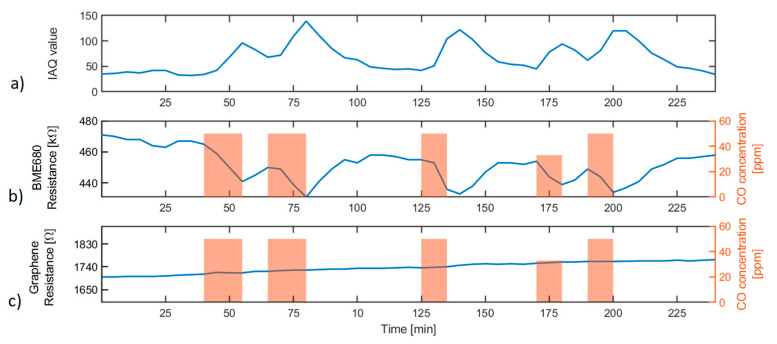
Sensor response to CO. (**a**) Index of air quality (IAQ) value calculated by means of the BME680 using the BSEC software library, (**b**) BME680 MOX sensor resistance, and (**c**) graphene sensor resistance. Right y-axes of graphs b and c show the CO concentration during the gas exposure.

Figure 9b,c show the responses from both commercial and lab-made sensors towards CO concentration changes during the simulation of gas leakage events and/or sudden increases in gas concentration. The index of air quality (IAQ) value was calculated by means of the BSEC software library provided by the manufacturer of the BME680 multisensor, which is shown in Figure 9a.

Results obtained for exposures to CO at 35 and 50 ppm during periods between 10 and 15 min show how the BME680 MOX sensor resistance and IAQ value follow the changes in gas concentration. In the same way, after every gas pulse, the BME680 sensor resistance starts recovering its initial value.

On the other hand, the graphene lab-made sensor resistance does not experience a significant variation during the exposures to CO. This does not mean that the sensor resistance remains constant, but the resolution of the resistance measurement channel, given by the analog-to-digital converter (ADC) resolution, is not high enough for detecting the very small resistance variations suffered by the graphene sensors in the presence of CO at the concentrations and pulse durations tested.

**Figure 10:**
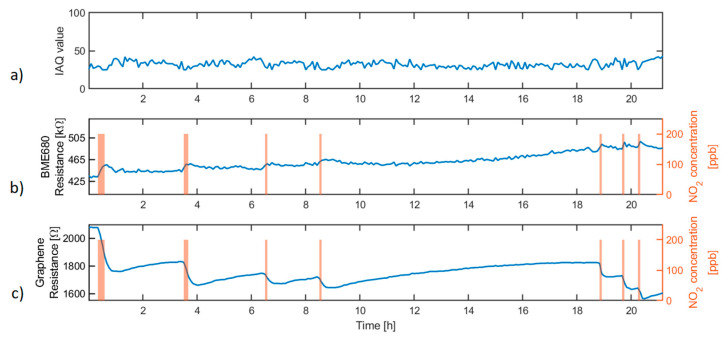
Sensor response to NO_2_. (**a**) IAQ value calculated by means of the Bosch Sensortec Environmental Cluster (BSEC) software library using the BME680 sensors exclusively, (**b**) BME680 metal oxide (MOX) sensor resistance, and (**c**) graphene sensor resistance. Right y-axes of graphs b and c show the NO_2_ concentration during the gas exposure.

Figure 10a shows the IAQ value obtained during the sensor response to NO_2_ pulses of 200 ppb applied during periods of 5, 10, and 15 min.

The results presented in Figure 10 show how although the BME680 sensor resistance slightly varies when the concentration of NO_2_ changes, the IAQ value does not reflect this variation, or even worse, it decreases in the presence of this pollutant gas. This is an expected result, as the BME680 is meant to be used for detecting VOCs and CO, which are reducing gases, while NO_2_ is an oxidising gas.

Conversely, the graphene lab-made sensor response to NO_2_ pulses is fully detectable by the readout circuitry implemented in the nodes. Resistance variations produced during the gas exposure have values of about 30 Ω for a 5-min exposure period. This variation is at least three times the noise background level and resistance drift when the sensor is exposed to the background air. This result makes the system suitable for the detection of a sudden increase in NO_2_ concentration.

The BME680 sensor is used as a general-purpose gas sensor through the IAQ value, with poor selectivity, and the IAQ value calculated is just negatively affected by reducing gases. The additional resistance measurement channel designed allows using a wide range of gas sensitive materials, thus giving selectivity and widening the set of target gases and applications where the sensor network can be applied. Further gas sensing response results can be found in Figure S3 (Supplementary Materials).

**Figure 11:**
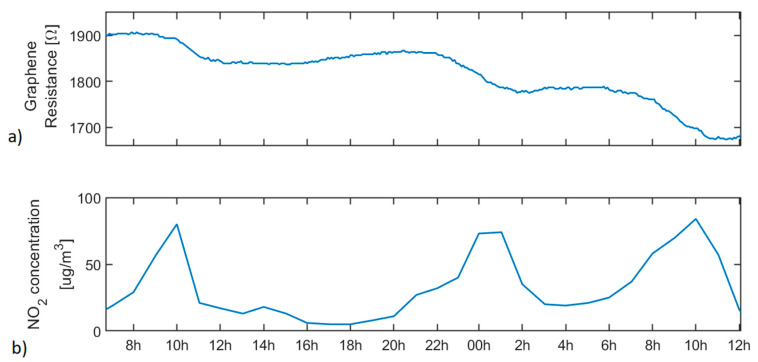
(**a**) Graphene sensor response during about 1 day of exposure to background level of NO_2_ present in ambient atmosphere, and (**b**) NO_2_ concentration registered by an automatic air quality station in Tarragona.

For longer exposure time analysis, the graphene sensor was left for about 1 day connected to the LoRa node (placed indoors) reacting with the NO_2_ background level present in the atmosphere. The sensor response was compared with the historical data registered by the automatic stations from the Network for the Surveillance and Forecast of Atmospheric Pollution in Catalonia obtained from [86]. Figure 11 shows the graphene sensor response towards the NO_2_ background level and the NO_2_ concentration registered every 1 h by the nearest air quality station to this node in Tarragona. The evolution of ambient humidity and temperature during this test is shown in Figure S4 (supporting information). This figure clearly shows that the changes experienced in humidity and temperature during this experiment did not affect the response of the graphene sensor. Two additional stations in Tarragona followed the same concentration trend during the same period analysed. Figure S5 in the supporting information shows the NO_2_ concentration data obtained from these stations. Finally, a 36 h continuous measurement experiment was conducted one month later than the one shown in Figure 11, and the response of the graphene sensor was compared again to the publicly available data for NO_2_ levels in the Tarragona area. These results are shown in Figure S8 (supporting information) and fully support the claim that the graphene sensor clearly indicates the episodes of high NO_2_ concentration in the ambient.

Despite the poor baseline recovery of graphene sensor due to the room temperature working conditions, it is worth noting that this gas sensor registered successive variations in its resistance when operated under real conditions experiments. These variations (registered indoors) correlate very well with episodes in which the outdoor NO_2_ concentration increased in the Tarragona area (as revealed by the data shown in Figure 11 and Figure S5). Therefore, in front of a sudden increase in the NO_2_ concentration level above the TLV, our system could easily detect it and raise an alarm.

If a quantitative rather than a qualitative analysis of nitrogen dioxide would be envisaged using the graphene sensor and the wireless network discussed here, the use of heating for periodically restoring the sensor surface and recovering its baseline would be needed. In addition, performing a calibration procedure would be needed as well. Figure S6 (supporting information) shows that the baseline recovery process for the graphene sensor is possible. In addition, Figure S7 (supporting information) shows a preliminary calibration curve towards NO_2_ in the range from 50 to 250 ppb obtained for the graphene sensor. These results indicate that our system shows potential for performing a quantitative analysis, but further work beyond the scope of this paper is necessary.

## 5. Conclusions

This work presents the development of a LoRa-based, scalable, low-cost, and low-power sensor network for air quality monitoring and gas leakage events detection in real time. The ability of the network of hot-plugging nodes allows the continuous operation and its easy scalability by just registering nodes on the cloud server application.

The commercial gas sensor used enables the detection of reducing gases such as VOCs and CO, and the calculation of an Index for Air Quality according to the total concentration of these pollutant species in the surroundings. However, this sensor is not able to give a proper IAQ value in the presence of oxidising pollutant gases, as the IAQ value decreases when the sensor reacts with NO_2_ (a decrease in the IAQ value is indicative of a better air quality and, obviously, this is not the case if the concentration of nitrogen dioxide increases). The limitations associated to the use of the IAQ value when an oxidising gas is present can be overcome using the lab-made graphene sensor. Graphene and the commercial sensors used in the sensor nodes development allow identifying the presence of pollutant reducing and oxidising gases through the IAQ value variation and the qualitative information obtained from the resistance variation of the graphene sensor, respectively. The graphene sensor developed not only enables the detection of sudden increases in NO_2_ concentrations but also makes the system suitable to follow the surrounding NO_2_ concentration trend in ambient air. The possibility of functionalising the graphene sensor presented here or the use of other gas sensitive materials can help modify the selectivity to different target gases, making this system suitable for being deployed in different application scenarios.

Despite the use of an inexpensive indoor gateway and 2 dBi omnidirectional antennas at the nodes, and having placed the nodes and gateway at different locations and distances, the RSSI values obtained were always above the sensitivity level, and the package loss rate was under 7% for every configuration tested.

The user-friendly web service developed for accessing the data remotely not only allows checking the values of the IAQ and the NO_2_ concentrations variation in a qualitative way, but it also shows the evolution of ambient humidity and temperature.

Further graphene functionalisation (e.g., nanoparticle decoration) is possible to enhance the results presented here. In addition, not limited to this, different gas sensing materials (e.g., MOXs or dichalcogenides) can be also adapted to the sensing nodes when other requirements are needed. This means that our sensing nodes present many possibilities to be improved and high versatility for being employed in different applications.

Future work will be focused in the quantitation of the target gases measured with chemoresistive sensors integrated in the sensing nodes. For example, by using heating for periodically restoring the sensor surface and recovering the baseline and by employing a calibration procedure, the graphene sensor could be used for quantitatively determining NO_2_ concentration.

## Figures and Tables

**Table 1:** Node communication performance. Received Signal Strength Indicator (RSSI) and package loss values according to nodes transmission power and distance between node and indoor gateway.

TP [dB]	Distance [m]	RSSI [dBm]	Package Loss [%]		
			Min.	Av.	Max.
12	10	−68	0	0	0
12	20	−84	0	0.34	0.68
12	115	−103	4.45	6.95	9.59
14	115	−101	3.82	5.09	6.6
20	130	−106	0.34	3.47	6.16

## Supplementary Materials

The following are available online at https://www.mdpi.com/1424-8220/20/21/6225/s1, Figure S1: Yearly number of publications related to (a) IoT, (b) IoT for air quality monitoring, (c) IoT using LoRa as wireless technology and (d) air quality monitoring; Figure S2: Coated alumina substrate; Figure S3: Graphene sensor response for NO_2_ and CO; Figure S4: Graphic interface of the web service for monitoring sensor data; Figure S5: Graphene sensor response during about 1 day of exposure to background level of NO_2_ present in ambient atmosphere, and NO_2_ concentration registered by 3 automatic air quality stations in Tarragona; Figure S6: Graphene sensor resistance baseline recovery process performed using temperature pulses; Figure S7: Calibration curve for the graphene sensor response at room temperature; Figure S8: Graphene sensor response during 36 h of exposure to background level of NO_2_ present in ambient atmosphere (upper panel) and NO_2_ concentration registered by four automatic air quality stations in the Tarragona area.

## References

[1] (2019). World Health Statistics 2019: Monitoring Health for the SDGs: Sustainable Development Goals.

[2] Kumar A., Singh I.P., Sud S.K. (2011). Energy efficient air quality monitoring system. Proc. IEEE Sens..

[3] Kim J.Y., Chu C.H., Shin S.M. (2014). ISSAQ: An integrated sensing systems for real-time indoor air quality monitoring. IEEE Sens. J..

[4] Chen Y.Y., Sung F.C., Chen M.L., Mao I.F., Lu C.Y. (2016). Indoor air quality in the metro system in north Taiwan. Int. J. Environ. Res. Public Health.

[5] Marques G., Pires I.M., Miranda N., Pitarma R. (2019). Air quality monitoring using assistive robots for ambient assisted living and enhanced living environments through internet of things. Electron.

[6] Serrano-Jiménez A., Lizana J., Molina-Huelva M., Barrios-Padura Á. (2020). Indoor environmental quality in social housing with elderly occupants in Spain: Measurement results and retrofit opportunities. J. Build. Eng..

[7] Farzad K., Khorsandi B., Khorsandi M., Bouamra O., Maknoon R. (2020). A study of cardiorespiratory related mortality as a result of exposure to black carbon. Sci. Total Environ..

[8] Ginsberg G.M., Kaliner E., Grotto I. (2016). Mortality, hospital days and expenditures attributable to ambient air pollution from particulate matter in Israel. Isr. J. Health Policy Res..

[9] Valentino S., Tarrade A., Chavatte-Palmer P. (2016). Exposition aux gaz d’échappement diesel durant la gestation: Quelles conséquences sur le développement fœto-placentaire? Apport des modèles animaux. Bull. Acad. Vet. Fr..

[10] Pan American Health Organization. www.paho.org/en/topics/air-quality.

[11] Chen X., Wang T., Qiu X., Que C., Zhang H., Zhang L., Zhu T. (2020). Susceptibility of individuals with chronic obstructive pulmonary disease to air pollution exposure in Beijing, China: A case-control panel study (COPDB). Sci. Total Environ..

[12] Effatpanah M., Effatpanah H., Jalali S., Parseh I., Goudarzi G., Barzegar G., Geravandi S., Darabi F., Ghasemian N., Mohammadi M.J. (2019). Hospital admission of exposure to air pollution in Ahvaz megacity during 2010–2013. Clin. Epidemiol. Glob. Health.

[13] World Health Organiaztion. www9.who.int/airpollution/en/.

[14] EPA Indoor Air Quality|EPA’s Report on the Environment (ROE)|US EPA. https://www.epa.gov/report-environment/indoor-air-quality.

[15] Wilbur S., Williams M., Williams R., Scinicariello F., Klotzbach J.M., Diamond G.L., Citra M. Toxicological profile for carbon monoxide. https://pubmed.ncbi.nlm.nih.gov/23946966/.

[16] EPA Basic Information about NO2|Nitrogen Dioxide (NO2) Pollution|US EPA. https://www.epa.gov/no2-pollution/basic-information-about-no2.

[17] EPA Quantitative Risk and Exposure Assessment for Carbon Monoxide—Amended. https://www3.epa.gov/ttn/naaqs/standards/co/data/CO-REA-Amended-July2010.pdf.

[18] EPA Volatile Organic Compounds’ Impact on Indoor Air Quality|Indoor Air Quality (IAQ)|US EPA. https://www.epa.gov/indoor-air-quality-iaq/volatile-organic-compounds-impact-indoor-air-quality.

[19] Maag B., Zhou Z., Thiele L. (2018). Monitoring Deployments. IEEE Internet Things J..

[20] Idrees Z., Zheng L. (2020). Low cost air pollution monitoring systems: A review of protocols and enabling technologies. J. Ind. Inf. Integr..

[21] Aberilla J.M., Gallego-Schmid A., Stamford L., Azapagic A. (2020). Environmental sustainability of cooking fuels in remote communities: Life cycle and local impacts. Sci. Total Environ..

[22] Ometov A., Bezzateev S., Voloshina N., Masek P., Komarov M. (2019). Environmental monitoring with distributed mesh networks: An overview and practical implementation perspective for urban scenario. Sensors.

[23] Brzozowski K., Maczyński A., Ryguła A. (2020). Monitoring road traffic participants’ exposure to PM10 using a low-cost system. Sci. Total Environ..

[24] Popoola O.A.M., Carruthers D., Lad C., Bright V.B., Mead M.I., Stettler M.E.J., Saffell J.R., Jones R.L. (2018). Use of networks of low cost air quality sensors to quantify air quality in urban settings. Atmos. Environ..

[25] Perez A.O., Bierer B., Scholz L., Wöllenstein J., Palzer S. (2018). A wireless gas sensor network to monitor indoor environmental quality in schools. Sensors.

[26] Prabakaran P., Manikandan A. (2019). Carbon monoxide air quality index (AQI-Co) and seasonal trend (ST-Co) in Chennai traffic zones, India. Int. J. Mech. Prod. Eng. Res. Dev..

[27] Polichetti T., Miglietta M.L., Alfano B., Massera E., De Vito S., Di Francia G., Faucon A., Saoutieff E., Boisseau S., Marchand N., Andò B., Baldini F., Di Natale C., Ferrari V., Marletta V., Marrazza G., Militello V., Miolo G., Rossi M., Scalise L. (2019). A Networked Wearable Device for Chemical Multisensing. Proceedings of the Sensors.

[28] Rickerby D.G., Skouloudis A.N. (2017). Nanostructured Metal Oxides for Sensing Toxic Air Pollutants. RSC Detection Science.

[29] Zappa D., Galstyan V., Kaur N., Munasinghe Arachchige H.M.M., Sisman O., Comini E. (2018). “Metal oxide -based heterostructures for gas sensors”—A review. Anal. Chim. Acta.

[30] Navarrete E., Bittencourt C., Umek P., Llobet E. (2018). AACVD and gas sensing properties of nickel oxide nanoparticle decorated tungsten oxide nanowires. J. Mater. Chem. C.

[31] Rodner M., Puglisi D., Ekeroth S., Helmersson U., Shtepliuk I., Yakimova R., Skallberg A., Uvdal K., Schütze A., Eriksson J. (2019). Graphene decorated with iron oxide nanoparticles for highly sensitive interaction with volatile organic compounds. Sensors.

[32] Oluwasanya P.W., Samad Y.A., Occhipinti L.G. Printable sensors for Nitrogen dioxide and Ammonia sensing at room temperature. Proceedings of the 2019 IEEE International Conference on Flexible and Printable Sensors and Systems (FLEPS).

[33] Sudhan N., Lavanya N., Leonardi S.G., Neri G., Sekar C. (2019). Monitoring of chemical risk factors for sudden infant death syndrome (SIDS) by hydroxyapatite-graphene-MWCNT composite-based sensors. Sensors.

[34] Peng H., Li F., Hua Z., Yang K., Yin F., Yuan W. (2018). Highly sensitive and selective room-temperature nitrogen dioxide sensors based on porous graphene. Sens. Actuators B Chem..

[35] Chen Y., Ghannam R., Heidari H. Air Quality Monitoring using Portable Multi-Sensory Module for Neurological Disease Prevention. Proceedings of the 2019 UK/China Emerging Technologies (UCET).

[36] Hussain S.A., Al Ghawi S., Al Rawahi B., Hussain S.J. (2020). Design and implementation of indoor environment monitoring and controlling system. Int. J. Adv. Sci. Technol..

[37] Chojer H., Branco P.T.B.S., Martins F.G., Alvim-Ferraz M.C.M., Sousa S.I.V. (2020). Development of low-cost indoor air quality monitoring devices: Recent advancements. Sci. Total Environ..

[38] Samee I.U., Jilani M.T., Wahab H.G.A. An Application of IoT and Machine Learning to Air Pollution Monitoring in Smart Cities. Proceedings of the 2019 4th International Conference on Emerging Trends in Engineering, Sciences and Technology (ICEEST).

[39] Zhao L., Wu W., Li S. (2019). Design and Implementation of an IoT-Based Indoor Air Quality Detector With Multiple Communication Interfaces. IEEE Internet Things J..

[40] Karunanithy K., Velusamy B. (2020). Energy efficient Cluster and Travelling Salesman Problem based Data Collection using WSNs for Intelligent Water Irrigation and Fertigation. Measurement.

[41] Saqib M., Almohamad T.A., Mehmood R.M. (2020). A low-cost information monitoring system for smart farming applications. Sensors.

[42] Difallah W., Bounaama F., Benahmed K., Draoui B., Maamar A. Smart irrigation technology for efficient water use. Proceedings of the 7th International Conference on Software Engineering and New Technologies.

[43] Lin R., Kim H.J., Achavananthadith S., Kurt S.A., Tan S.C.C., Yao H., Tee B.C.K., Lee J.K.W., Ho J.S. (2020). Wireless battery-free body sensor networks using near-field-enabled clothing. Nat. Commun..

[44] Geman O., Chiuchisan I., Ungurean I., Hagan M., Arif M. (2018). Ubiquitous healthcare system based on the sensors network and android internet of things gateway. Proceedings of the 2018 IEEE SmartWorld, Ubiquitous Intelligence & Computing, Advanced & Trusted Computed, Scalable Computing & Communications, Cloud & Big Data Computing, Internet of People and Smart City Innovation (SmartWorld/SCALCOM/UIC/ATC/CBDCom/IOP/SCI).

[45] Chatterjee A., Biswas J., Das K. (2020). An automated patient monitoring using discrete-time wireless sensor networks. Int. J. Commun. Syst..

[46] Gautam S.K., Om H. (2018). Intrusion detection in RFID system using computational intelligence approach for underground mines. Int. J. Commun. Syst..

[47] Almomani I., Alromi A. (2020). Integrating software engineering processes in the development of efficient intrusion detection systems in wireless sensor networks. Sensors.

[48] Vladuta V.A., Apostol I., Ghimes A.M. Data Collection Analysis: Field Experiments with Wireless Sensors and Unmanned Aerial Vehicles. Proceedings of the 2018 International Conference on Communications (COMM).

[49] Landaluce H., Arjona L., Perallos A., Falcone F., Angulo I., Muralter F. (2020). A review of iot sensing applications and challenges using RFID and wireless sensor networks. Sensors.

[50] Awadallah S., Moure D., Torres-González P. (2019). An internet of things (IoT) application on volcano monitoring. Sensors.

[51] Villa-Henriksen A., Edwards G.T.C., Pesonen L.A., Green O., Sørensen C.A.G. (2020). Internet of Things in arable farming: Implementation, applications, challenges and potential. Biosyst. Eng..

[52] Chang C., Srirama S.N., Buyya R. (2019). Internet of Things (IoT) and New Computing Paradigms. Fog Edge Comput..

[53] Xia F., Yang L.T., Wang L., Vinel A. (2012). Internet of things. Int. J. Commun. Syst..

[54] Zhou H., Li S., Chen S., Zhang Q., Liu W., Guo X. (2020). Enabling Low Cost Flexible Smart Packaging System with Internet-of-Things Connectivity via Flexible Hybrid Integration of Silicon RFID Chip and Printed Polymer Sensors. IEEE Sens. J..

[55] Devan P.A.M., Hussin F.A., Ibrahim R., Bingi K., Nagarajapandian M. IoT Based Vehicle Emission Monitoring and Alerting System. Proceedings of the 2019 IEEE Student Conference on Research and Development (SCOReD).

[56] Rustia D.J.A., Lin C.E., Chung J.Y., Zhuang Y.J., Hsu J.C., Lin T. (2020). Te Application of an image and environmental sensor network for automated greenhouse insect pest monitoring. J. Asia Pac. Entomol..

[57] Rodríguez-Robles J., Martin Á., Martin S., Ruipérez-Valiente J.A., Castro M. (2020). Autonomous sensor network for rural agriculture environments, low cost, and energy self-charge. Sustainability.

[58] Chaudhari B.S., Zennaro M., Borkar S. (2020). LPWAN technologies: Emerging application characteristics, requirements, and design considerations. Futur. Internet.

[59] (2015). LoRa Alliance White Paper: A Technical Overview of LoRa and LoRaWAN. https://lora-alliance.org/resource-hub/what-lorawanr.

[60] (2017). LoRa Alliance Technical Commitee LoRaWAN 1.1 Specification. https://lora-alliance.org/resource-hub/lorawanr-specification-v11.

[61] Van Wijnen J.H., Verhoeff A.P., Jans H.W.A., van Bruggen M. (1995). The exposure of cyclists, car drivers and pedestrians to traffic-related air pollutants. Int. Arch. Occup. Environ. Health.

[62] Novikov S., Lebedeva N., Satrapinski A., Walden J., Davydov V., Lebedev A. (2016). Graphene based sensor for environmental monitoring of NO2. Sens. Actuators B Chem..

[63] Panda D., Nandi A., Datta S.K., Saha H., Majumdar S. (2016). Selective detection of carbon monoxide (CO) gas by reduced graphene oxide (rGO) at room temperature. RSC Adv..

[64] Sensortec B. (2019). BME680 Low Power Gas, Pressure, Temperature & Humidity Sensor. https://cdn-shop.adafruit.com/product-files/3660/BME680.pdf.

[65] Lozano J., Suarez J.I., Melendez F., Rodriguez S., Arroyo P., Herrero J.L., Carmona P. (2019). Personal electronic systems for citizen measurements of air quality. Proceedings of the 2019 5th Experiment at International Conference (exp.at 2019).

[66] Jose J., Sasipraba T. (2019). Indoor air quality monitors using IOT sensors and LPWAN. Proceedings of the International Conference on Trends in Electronics and Informatics (ICOEI 2019).

[67] Lasomsri P., Yanbuaban P., Kerdpoca O., Ouypornkochagorn T. (2019). A development of low-cost devices for monitoring indoor air quality in a large-scale hospital. Proceedings of the ECTI-CON 2018—15th International Conference on Electrical Engineering/Electronics, Computer, Telecommunications and Information Technology.

[68] Thakor G.S.N., Hu P., Motisan R., Chiang E., Santos R. Testing & validation of mobile air quality monitor for sensing & dilineating VOC emissions. Proceedings of the Air and Waste Management Association’s Annual Conference and Exhibition.

[69] Eklund P.C., Holden J.M., Jishi R.A. (1996). Vibrational Modes of Carbon Nanotubes; Spectroscopy and Theory.

[70] Wu J.B., Lin M.L., Cong X., Liu H.N., Tan P.H. (2018). Raman spectroscopy of graphene-based materials and its applications in related devices. Chem. Soc. Rev..

[71] Jorio A. (2012). Raman Spectroscopy in Graphene-Based Systems: Prototypes for Nanoscience and Nanometrology. ISRN Nanotechnol..

[72] Yang G., Kim B.-J., Kim K., Woo Han J., Kim J. (2015). Energy and dose dependence of proton-irradiation damage in graphene. RSC Adv..

[73] Acosta S., Casanova Chafer J., Sierra Castillo A., Llobet E., Snyders R., Colomer J.-F., Quintana M., Ewels C., Bittencourt C. (2019). Low Kinetic Energy Oxygen Ion Irradiation of Vertically Aligned Carbon Nanotubes. Appl. Sci..

[74] Ganesan K., Ghosh S., Gopala Krishna N., Ilango S., Kamruddin M., Tyagi A.K. (2016). A comparative study on defect estimation using XPS and Raman spectroscopy in few layer nanographitic structures. Phys. Chem. Chem. Phys..

[75] Ma J., Zhang M., Dong L., Sun Y., Su Y., Xue Z., Di Z. (2019). Gas sensor based on defective graphene/ pristine graphene hybrid towards high sensitivity detection of NO2. AIP Adv..

[76] Casanova-Cháfer J., García-Aboal R., Atienzar P., Llobet E. (2019). Gas Sensing Properties of Perovskite Decorated Graphene at Room Temperature. Sensors.

[77] Casanova-Cháfer J., Navarrete E., Noirfalise X., Umek P., Bittencourt C., Llobet E. (2018). Gas Sensing with Iridium Oxide Nanoparticle Decorated Carbon Nanotubes. Sensors.

[78] Prakash A., Majumdar S., Devi P.S., Sen A. (2009). Polycarbonate membrane assisted growth of pyramidal SnO_2_ particles. J. Memb. Sci..

[79] Shankar P., Rayappan J. (2015). Gas sensing mechanism of metal oxides: The role of ambient atmosphere, type of semiconductor and gases-A review. Sci. Lett. J..

[80] Liu X.Y., Zhang J.M., Xu K.W., Ji V. (2014). Improving SO2 gas sensing properties of graphene by introducing dopant and defect: A first-principles study. Appl. Surf. Sci..

[81] Zhang Y.H., Chen Y.B., Zhou K.G., Liu C.H., Zeng J., Zhang H.L., Peng Y. (2009). Improving gas sensing properties of graphene by introducing dopants and defects: A first-principles study. Nanotechnology.

[82] Deokar G., Casanova-Cháfer J., Rajput N.S., Aubry C., Llobet E., Jouiad M., Costa P.M.F.J. (2020). Wafer-scale few-layer graphene growth on Cu/Ni films for gas sensing applications. Sens. Actuators B Chem..

[83] Knight S., Hofmann T., Bouhafs C., Armakavicius N., Kühne P., Stanishev V., Ivanov I.G., Yakimova R., Wimer S., Schubert M. (2017). In-situ terahertz optical Hall effect measurements of ambient effects on free charge carrier properties of epitaxial graphene. Sci. Rep..

[84] Standards—Air Quality—Environment—European Commission. https://ec.europa.eu/environment/air/quality/standards.htm.

[85] NAAQS Table|Criteria Air Pollutants|US EPA. https://www.epa.gov/criteria-air-pollutants/naaqs-table.

[86] Descàrrega de dades Departament de Territori i Sostenibilitat. http://mediambient.gencat.cat/ca/05_ambits_dactuacio/atmosfera/qualitat_de_laire/vols-saber-que-respires/descarrega-de-dades/.

