# Supplementary material for: LoRa Sensor Network Development for Air Quality Monitoring or Detecting Gas Leakage Events
Source: Sensors (Basel). 2020 Oct 31;20(21):6225. doi: 10.3390/s20216225 (PMC7672618; doi:10.3390/s20216225)
Supplement: Supplementary file 1 [file sensors-20-06225-s001.docx]

Supporting Information

LoRa Sensor Network development for Air Quality Monitoring or Detecting Gas Leakage Events

Ernesto González ^1^, Juan Casanova-Chafer ^1^, Alfonso Romero ^1^, Xavier Vilanova ^1,*^, Jan Mitrovics ^2^, and Eduard Llobet ^1^

^1^ Uiversitat Rovira i Virgili, MINOS; Tarragona, Spain

^2^ JLM Innovation GmbH, Tubingen, Germany; jan.mitrovics@jlm-innovation.de

***** Correspondence: xavier.vilanova@urv.cat; Tel.: +34-977 558 502 (ES)

Figure S1 shows the growth of the research areas for which this paper is relevant, reflected as the number of publications appearing in Scopus between years 2010 and 2020 related to IoT, LoRa, and air quality monitoring.


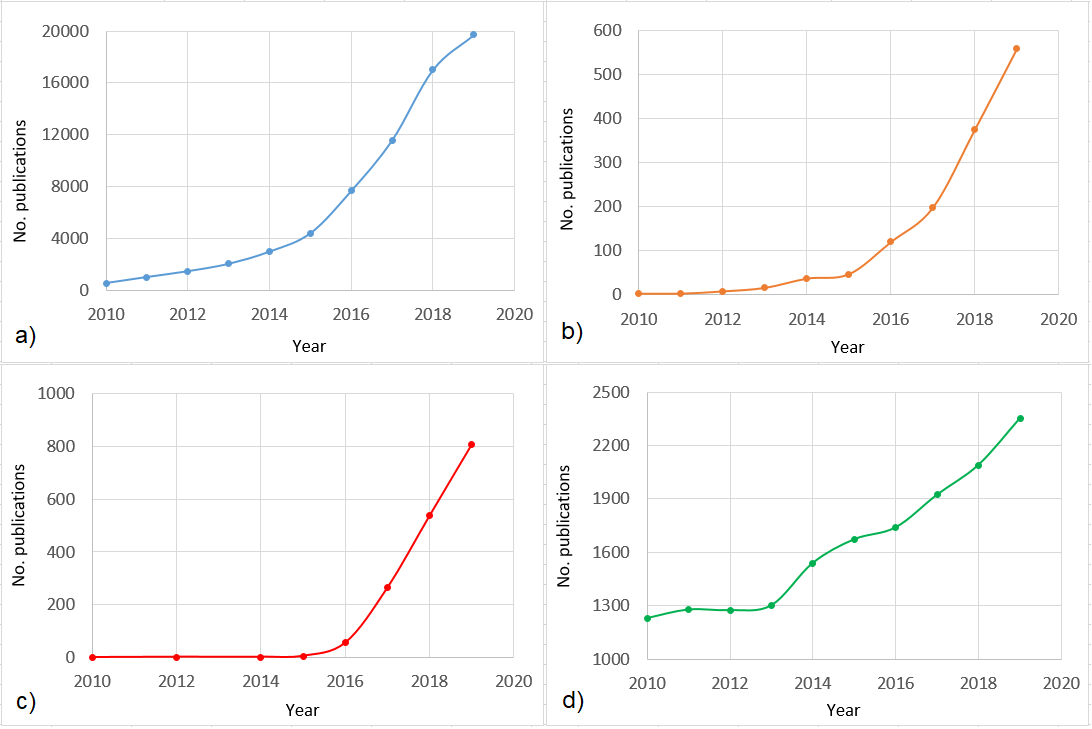


**Figure S1.** Yearly number of publications related to (a) IoT, (b) IoT for air quality monitoring, (c) IoT using LoRa as wireless technology and (d) air quality monitoring. Source: Scopus.

Graphene sensor


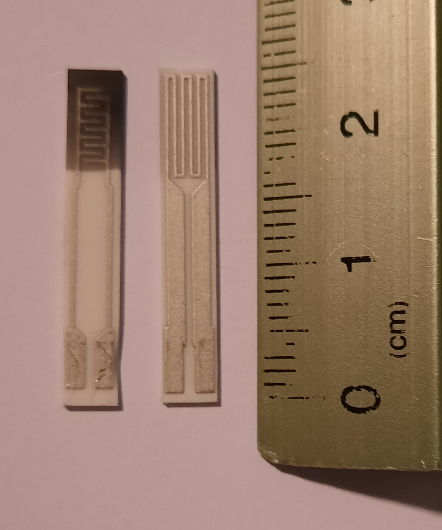


**Figure S2**. Alumina substrate. Top side (left) with graphene nanoplatelets deposited on the interdigitated electrodes. Bottom side (right) with a platinum screen printed heater.

Graphene sensor performance

In order to test the in-lab performance of the graphene sensor towards CO and NO_2_, preliminary measurements were done under controlled atmosphere. During the laboratory conditions test sensors were placed in a Teflon chamber with an inner volume of about 22 cm^3^. NO_2_ and CO calibrated cylinders of 1 ppm and 100 ppm respectively (balanced in synthetic air), and synthetic air cylinders were used to set the desired gas concentrations by using a mass-flow controller system (EL-FLOW®) from Bronkhorst, and Flow View and Flow Plot software from the same company were employed. The total flow across the sensor chamber was kept at 100 ml/min. As the resistance measurements channel was meant to be used to detect resistance changes provoked by gas leakage events and sudden increases in gas concentrations, sensors were tested with two different experiments. First, a gas leakage event was simulated (Figure S3(a) and S3(c)) by exposing the sensor to a period under synthetic air and then a short exposure of 15 min under gas concentration above the Threshold Limit Value (TLV). On the other hand, for simulating sudden increases in gas concentration sensors were exposed to low concentrations of target gases (below the TLV) and then to a period of 15 min of concentrations above the TLV (Figure S3(b) and S3(d)). Gas concentrations used were 35 ppm and 200 ppb for CO and NO_2,_ respectively. These concentrations were selected according to the exposure limits for 1 h established by the European Environment Agency and the National Ambient Air Quality Standards from the United States Environmental Protection Agency. Figure S3 shows the response of the graphene sensor during the test mentioned above.


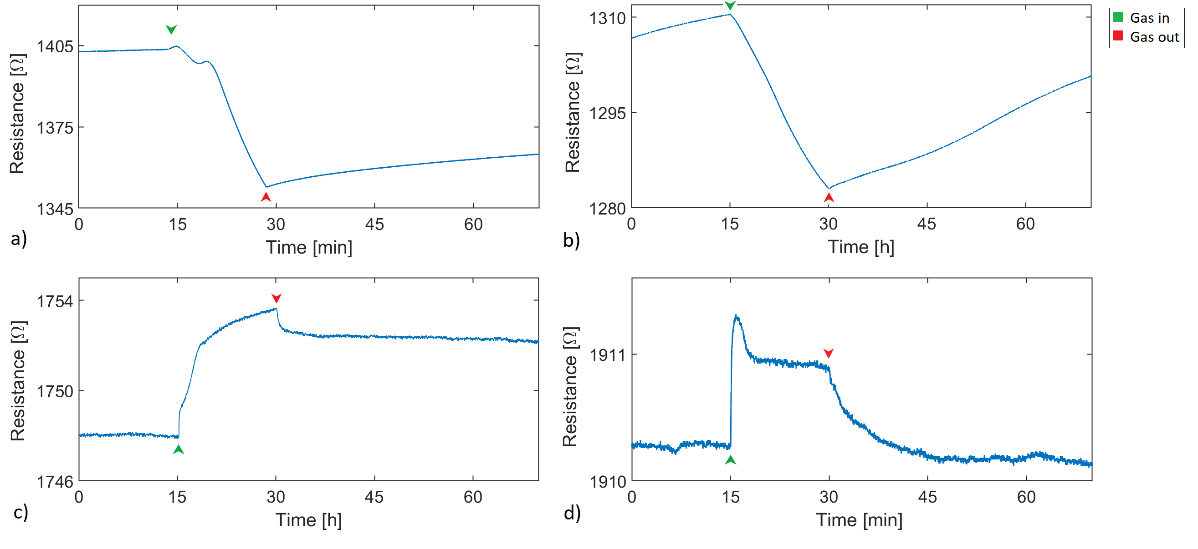


**Figure S3.** Graphene sensor response for (a) 15 min of 200 ppb of NO_2_ and then synthetic air, (b) baseline NO_2_ level of 50 ppb (below the TLV) and sudden increase to 200 ppb during 15 minutes (overpassing the TLV), (c) 35 ppm of CO and then a period under synthetic air, and (d) baseline CO level of 5 ppm and sudden increase to 35 ppm during 15 min. (a) and (c) correspond to the simulation of a gas leakage event where gas concentrations variate from 0 to values above the TLV, while (b) and (d) simulate a sudden increase in gas concentration from values below the TLV to values over this.

To accomplish the purpose of detecting gas leakage events or sudden increases in gas concentrations, the resistance measurement channel must be able to detect the resistance variation (decrease for NO_2_ and increase for CO) caused by the sensor response towards the interaction with gases. Since the nodes are configured to perform the resistance calculation using a 12-bit analog-to-digital converter (ADC), and the V_DD_ of the system is 3.3 V, the resolution is 8.0566 x10^-4^ V. Thus, any change in resistance that provokes a voltage variation higher than this value would be detected by the system. Considering that the resistance variation results presented in Figure S3a,b,c provoked by the interaction of both, NO_2_ and CO with the sensor generate a voltage variation higher than the ADC resolution, the resistance measurement channel is able to operate in these scenarios. On the other hand, when CO concentration goes from 5 ppm to 35 ppm, represented in Figure S3d, resistance variation corresponds to a voltage change lower than the ADC resolution. Hence, this increase in resistance is not detectable by the system.

Therefore, a sudden increase (from a background level to a value above the TLV) in CO concentration would not probably be detected. Besides, despite the theoretical detection of CO in gas leakage events (Figure S3c) in-lab conditions, the graphene sensor is not able to detect this variation from 0 to 35 ppm of CO during ambient monitoring as Figure 9c shows in the manuscript. Probably the reason is based on the ambient moisture conditions. In other words, while Figure S3 shows in-lab measurement under dry conditions, Figure 9 in the manuscript represents the real time-monitoring, in which the atmosphere contains a significant level of relative humidity. In consequence, as the gas sensing mechanisms explained in the manuscript, the electron-withdrawing behavior of water molecules could underestimate the CO response, being almost impossible to detect this analyte even in gas leakage events.

Nodes and Gateway Cost

As the total cost of the network depends on the number of nodes deployed, the cost presented here is just related to one gateway and one node. The price of the TTIG (gateway) at the moment of starting the project was 69 euros. Nodes parts and components have a price of 39.43 euros including the LoRa development board and 2 dBi omnidirectional antenna, BME680 4-in-1 sensor, PCB and electronic components and 3D printed package.

Qualitative background NO_2_ concentration variation measurement


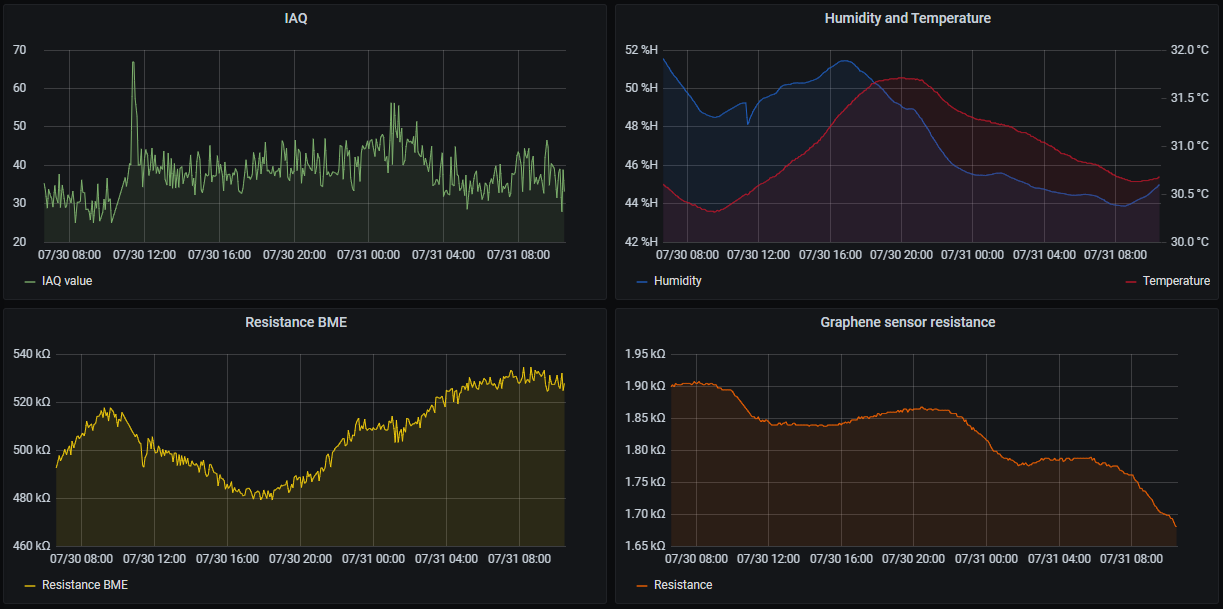


**Figure S4.** Graphic interface of the web service for monitoring sensor data. IAQ value calculated from the BME680 sensor (top-left), temperature (top-right), humidity (top-right), BME680 sensor resistance (bottom-left), and graphene lab-synthesized sensor resistance (bottom-right). The figure shows the same time period than Figure 11 in the manuscript.

As Figure S4 shows while the BME680 sensor resistance somehow follows the temperature and humidity changes, the graphene lab-synthesized sensor resistance does not show the same behavior. Thus, it can be deduced that the graphene sensor resistance is just being affected by the background NO_2_ concentration. This supports the results presented in Figure 11 in section **4.2. Gas sensing performance**. The peak appearing in the IAQ value chart is related to a small pulse of 35 ppm of CO applied before letting the sensor to react just with the ambient air.


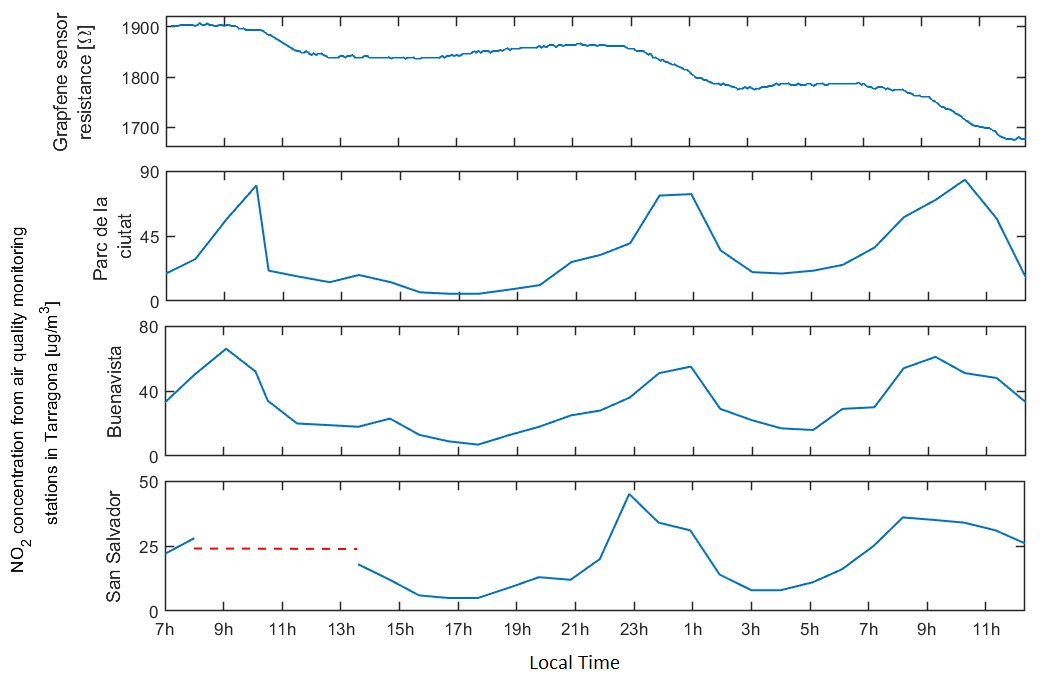


**Figure S5**. Graphene sensor response during about 1 day of exposure to background level of NO_2_ present in ambient atmosphere, and NO_2_ concentration registered by 3 automatic air quality stations in Tarragona. Distance from the stations to the sensor nodes location was about 1.75 km (Parc de la ciutat), 4.7 km (Bonavista), and 2.9 km (Sant Salvador). The information related to the spacetime in which Sant Salvador plot appears with a red dotted line is not available in the web service form the Generalitat de Catalunya.

Figure S5 depicts how although the NO_2_ concentration along the 3 automatic stations have different levels, the concentration trend is the same. This support the results presented in Figure 11 in the manuscript.

Baseline recovery and sensor calibration


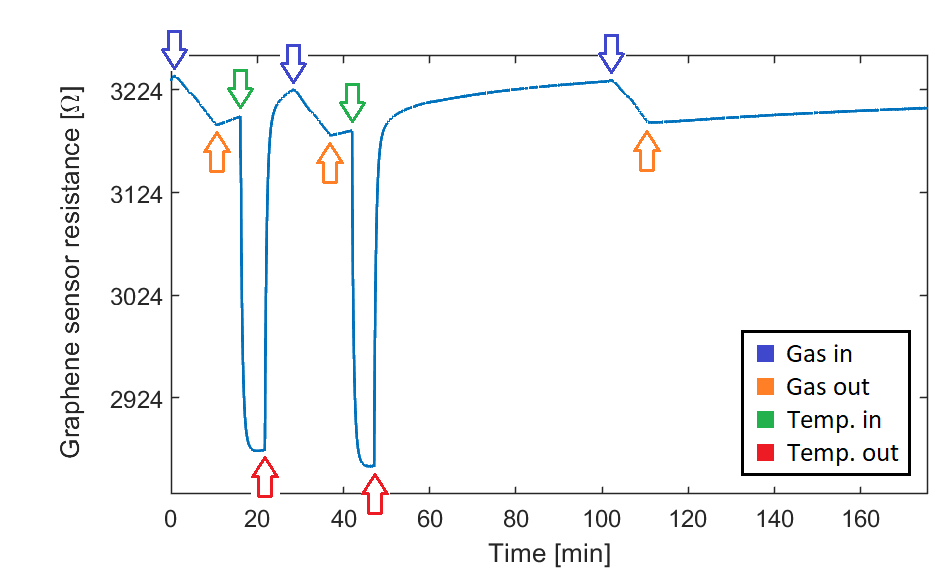


**Figure S6**. Graphene sensor resistance baseline recovery process performed using temperature pulses.

Figure S6 depicts the baseline recovery process for the graphene sensor resistance. This test was performed by applying 250 ppb NO_2_ pulses during about 10 min, after which the sensor is exposed to synthetic air. After being 5 min under synthetic air, a temperature pulse of 50ºC is applied for 5 min under synthetic air to restore the sensor surface. After the final NO_2_ pulse the temperature pulse is not applied to show how the resistance baseline is not recovered at room temperature even after 1 h.


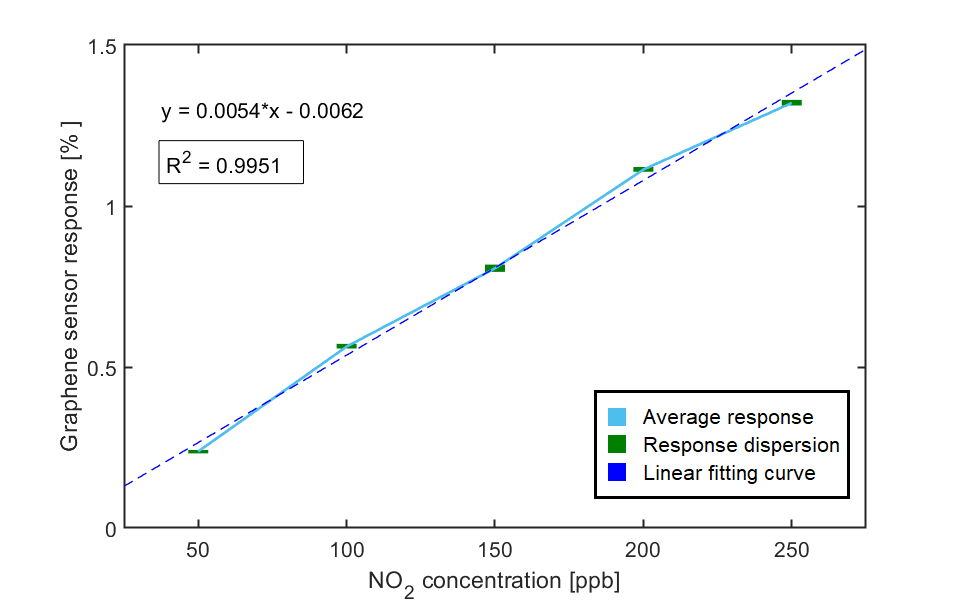


**Figure S7**. Calibration curve for the graphene sensor response at room temperature.

Additional in-lab measurements have been carried out by using the graphene sensor to assess the sensing performance to NO_2_. Therefore, calibrated gas bottles and a mass flow system were used to apply successive concentrations of NO_2_ for 5 minutes, followed by 15 minutes of synthetic dry air between the different gas exposures, in order to desorb the NO_2_ molecules and recover the resistance baseline (flow rate: 400 sccm). Figure S7 shows the calibration curve obtained for a range of NO_2_ concentrations from 50 ppb to 250 ppb. A quite linear gas sensor response was obtained, as well as high measurement repeatability.


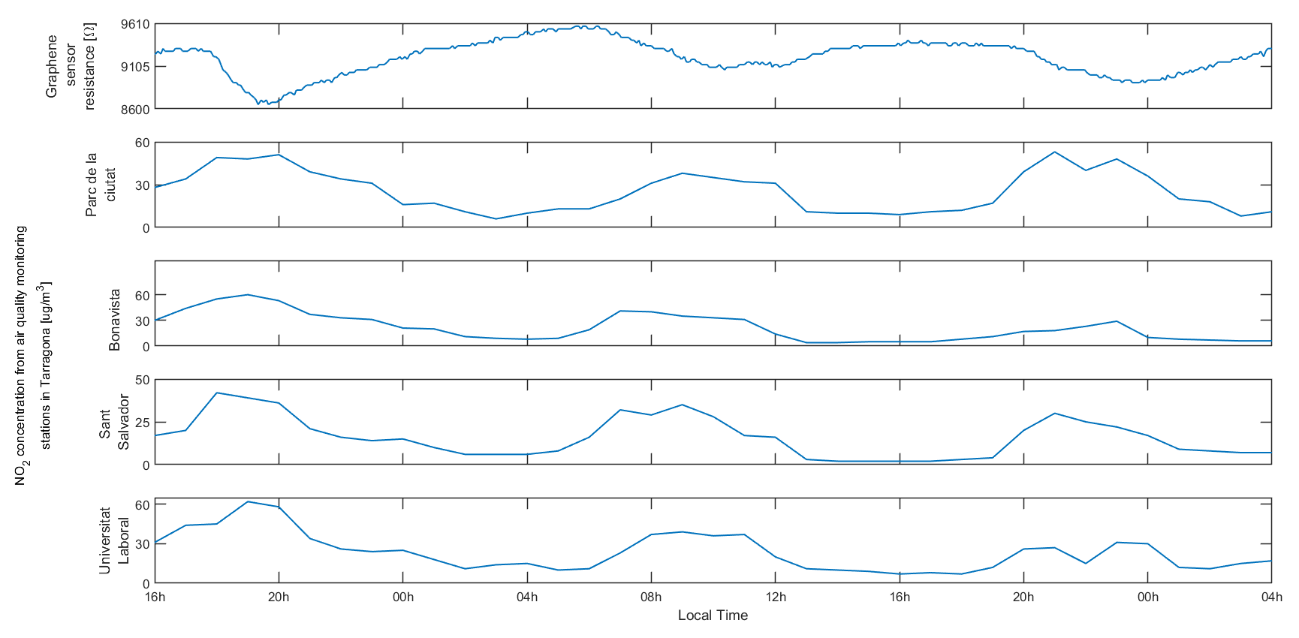


**Figure S8.** Graphene sensor response during 36 h of exposure to background level of NO_2_ present in ambient atmosphere (upper panel) and NO_2_ concentration registered by four automatic air quality stations in the Tarragona area.

This experiment further confirms that the graphene sensor is able to clearly follow and indicate the episodes in which the background ambient levels of nitrogen dioxide increase.
